# Biography: Shashank Shah, MBBS, MS, FAIS, FICS

**DOI:** 10.1007/s11695-020-05138-3

**Published:** 2021-01-06

**Authors:** Shashank Shah

**Affiliations:** Department of Laparoscopic and Bariatric Surgery, Laparo-Obeso Centre, 2143, Sadashiv Peth, Vijayanagar Colony, Near Neelayam Talkies, Pune, Maharashtra 411030 India

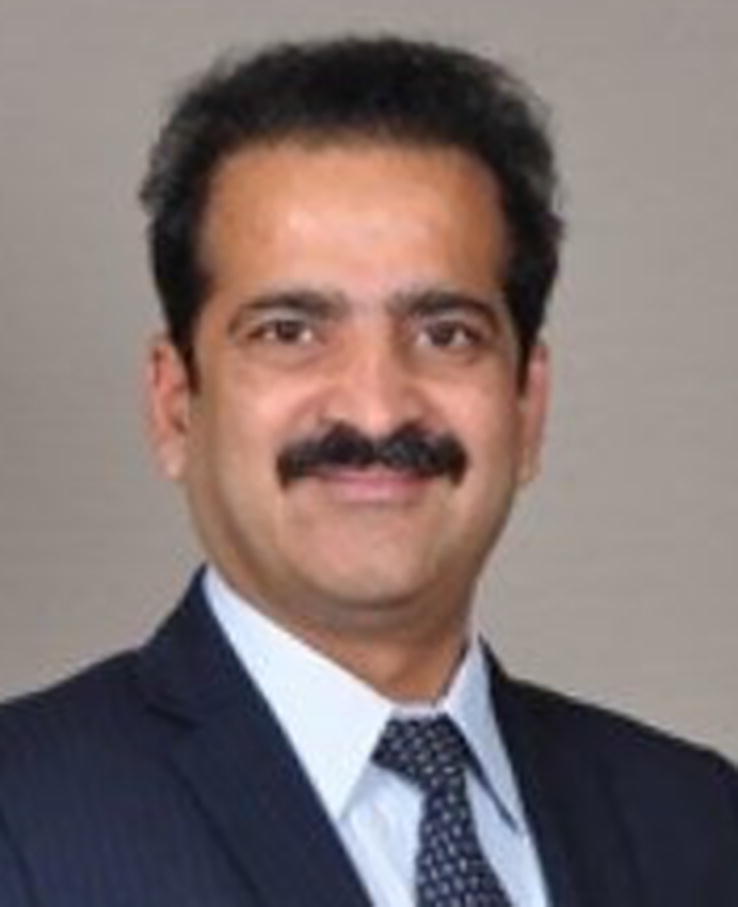



Shashank Shah, MBBS, MS, FAIS, FICS, is an Indian pioneer in laparoscopic bariatric surgery. He holds numerous titles such as Honorary FIAGES, Honorary FMAS, Honorary FMBS, and Honorary FALS. Dr. Shah completed his fellowship in bariatric surgery at many facilities worldwide including the USA, Italy, France, Brazil, and various other renowned centers across the world. He also has a Fellowship in Minimally Access Surgery (FMAS). Dr. Shah was also awarded an Honorary Fellowship in Metabolic and Bariatric Surgery (FMBS) from OSSICON in 2020.

Dr. Shah is the Director of the Laparo Obeso Centre, one of Asia’s highest volume centers for bariatric surgery. He is also a consultant to some of the leading hospitals in India such as Lilavati Hospital and Research Centre and Hinduja Healthcare. He is the Head of the Department for Bariatric and Metabolic Surgery at Poona Hospital and Research Centre, was the only Indian faculty member awarded a Diploma as a visiting professor for metabolic and bariatric surgery in the world-renowned university, IRCAD in France, and is also a visiting professor for several Malaysian universities.

Dr. Shah strives for excellence in surgery. He has extensive experience in the challenging field of metabolic and bariatric surgery and has worked to expand bariatric surgery to reach more Indians and Asians who are suffering from metabolic diseases like diabetes with obesity.

Dr. Shah is the first surgeon ever to receive the prestigious American Diabetes Association’s Vivian Fonseca Scholar Award in 2016. He was recognized for this honor during the Association’s 76th Scientific Sessions in New Orleans, USA. Dr. Shah received this Award for his study, “Gastric Bypass vs. Medical/Lifestyle Care for Type 2 Diabetes in South Asians with BMI 25-40 kg/m^2^—The COSMID Randomized Trial,” for which he was the Principal Investigator.

Recently, Dr. Shah was recognized by the World Book of Records, Limca Book of Records, and the India Book of Records for operating on the heaviest woman in the world who weighed 300 kg. Four years after surgery, she has maintained her weight at 86 kg. He also was recognized in the Limca book of records in 2005 for setting a world record by performing 45 hernia surgeries in just 10 h.

Dr. Shashank Shah is also recognized for successfully performing high-risk surgeries. He has performed bariatric surgery on one of the youngest boys (7.5 years) in the world and also on one of the heaviest British (310 kg). The BBC channel recognized his unique work by recording two special documentaries with him. The National Geographic Channel also recognized his work and dedication to prevent childhood and adolescent obesity by recording an awareness documentary with Dr. Shah about an Indian child who had become an obese adolescent.

Dr. Shashank Shah is an innovator and has developed a less invasive thoracoscopic procedure for treating myasthenia gravis than the standard technique of thoracoscopic radical thymectomy. It was published in the international reference video library of the Society of American Gastrointestinal and Endoscopic Surgeons (SAGES). This technique has been recognized by the National Neurological Society for being less invasive than the standard radical thymectomy.

Dr. Shah has also received the prestigious IIT Innovations Award for his research in “Surgery for Type 2 Diabetes” at the IIT Mumbai.

Dr. Shah is the first Indian bariatric surgeon to have published multiple papers in indexed journals on the impact of bariatric surgery on Asians, including Indians. In 2009, he published a paper on the mechanistic and physiological impact of the sleeve gastrectomy titled, “Decreased small bowel transit time (SBTT) after sleeve gastrectomy (SG): Possible early ileal stimulation as an additional proposed mechanism of action for type 2 diabetes (T2DM) resolution.” Furthermore, he conducted some of the earliest research on the impact of gastric bypass on low BMI patients with T2DM. He published the results in a paper titled, “Should gastric bypass operations be done for type 2 diabetes in subjects with body mass index 20 – 34 kgs/m 2? - An initial Indian experience.” In addition, he wrote the first paper on the role of the gut microbiota in obese patients and the change after bariatric surgery titled, “Molecular analysis of gut microbiota in obesity among Indian individuals.”

Recognizing Dr. Shah’s knowledge and research in the field of type 2 diabetes, he was the first international speaker invited to participate in the Innovations in Surgery, a video conference of the prestigious Cleveland Clinic, USA. His presentation was relayed to 36 universities globally. This presentation resulted in Dr. Shah being the first Indian to participate in the Consensus for Sleeve Gastrectomy Summit to form safe practice guidelines for surgeons worldwide.

Dr. Shah was the first Indian to be elected as the president of three national societies at the same time—namely, All India Association for Advancing Research in Obesity (AIAARO), Obesity Surgery Society of India (OSSI), and IEF (International Excellence Federation, India Chapter).

Considering the increasing obese population of India and an increasing demand for bariatric surgeons in India, Dr. Shah started the first laparoscopic bariatric training center in India and has so far trained and proctored over 2000 surgeons including Indians and international surgeons. Dr. Shah has also traveled across the country proctoring, training, and establishing hundreds of centers for the treatment of obesity and diabetes.

In his 30 years as a surgeon, Dr. Shah has performed over 40,000 surgical procedures and over 7000 total bariatric procedures.

Dr. Shah has served people by working as one of the few versatile surgeons in India who could perform general surgery, laparoscopic surgery, hernia surgery, and also laparoscopic bariatric and metabolic surgery. In addition to all the above, Dr. Shashank Shah has been offering his expert services to the poor and needy as well as in charitable institutes all over the country.

Dr. Shah is married to the pioneer Bariatric Physician, Dr. Poonam Shah, who has been his support system for over 30 years. Dr. Poonam is an active researcher and is also involved in many organizations like IFSO, ASMBS, and IASO. They have a beautiful pair of twins who are also studying to be doctors. In his leisure time, Dr. Shashank Shah enjoys singing, traveling, and spending time with his family.

